# Molecular Phylogeny and Ecology of *Textularia agglutinans* d’Orbigny from the Mediterranean Coast of Israel: A Case of a Successful New Incumbent

**DOI:** 10.1371/journal.pone.0142263

**Published:** 2015-11-05

**Authors:** Gily Merkado, Danna Titelboim, Orit Hyams-Kaphzan, Maria Holzmann, Jan Pawlowski, Ahuva Almogi-Labin, Uri Abdu, Barak Herut, Sigal Abramovich

**Affiliations:** 1 Ben Gurion University of the Negev, Beer Sheva, Israel; 2 Geological Survey of Israel, Jerusalem, Israel; 3 Department of Genetics and Evolution, University of Geneva, Genève, Switzerland; 4 Israel Oceanographic and Limnological Research, National Institute of Oceanography, Haifa, Israel; University of Otago, New Zealand, NEW ZEALAND

## Abstract

*Textularia agglutinans* d’Orbigny is a non-symbiont bearing and comparatively large benthic foraminiferal species with a widespread distribution across all oceans. In recent years, its populations have considerably expanded along the Israeli Mediterranean coast of the eastern Levantine basin. Despite its exceptionally widespread occurrence, no molecular data have yet been obtained. This study provides the first ribosomal DNA sequences of *T*. *agglutinans* complemented with morphological and ecological characterization, which are based on material collected during environmental monitoring of the hard bottom habitats along the Israeli Mediterranean coast, and from the Gulf of Elat (northern Red Sea). Our phylogenetic analyses reveal that all specimens from both provinces belong to the same genetic population, regardless their morphological variability. These results indicate that modern population of *T*. *agglutinans* found on the Mediterranean coast of Israel is probably Lessepsian. Our study also reveals that *T*. *agglutinans* has an epiphytic life mode, which probably enabled its successful colonization of the hard bottom habitats, at the Mediterranean coast of Israel, which consist of a diverse community of macroalgae. Our study further indicates that the species does not tolerate high SST (> 35°C), which will probably prevent its future expansion in the easternmost Mediterranean in light of the expected rise in temperatures.

## Introduction

*Textularia agglutinans* d’Orbigny is a large cosmopolitan agglutinated foraminifera species, with an elongated biserial test and a low arch aperture. It was first described by d’Orbigny in 1839 [1] from sandy beaches in Cuba, (type locality was not designated) and has been reported since from numerous locations worldwide. These include: Atlantic Ocean [2–6], Red Sea [7], Timor Sea [8], Pacific Ocean [9–13], Indian Ocean [14–16], Western and Eastern Mediterranean [17–30], Adriatic Sea[31–33], Tyrrhenian Sea [28], and Marmara Sea [27].

The ecological observations suggested that *T*. *agglutinans* generally favors sandy or muddy bottom sediment with a certain preference for a low input of clay [31,34]. In some cases, specimens were reported as infaunal with a preference for the uppermost oxygenated sediment layer [22].

Despite its common occurrences in all oceans, the phylogeny and genetic diversity of *T*. *agglutinans*, have not yet been examined. This study was motivated by the apparent recent expansion of *T*. *agglutinans* in the Israeli Mediterranean shelf [21], were it is found from very shallow depths of several centimeters [26] up to 120 meters [18,35]. Our study provides new insights on the source of the living population of *T*. *agglutinans* in the Israeli Mediterranean shelf, as well as on the species phylogeny and ecology by combining genetic investigation with quantitative faunal data and high-resolution temperature records.

## Methods

### Study area

#### Mediterranean coast of Israel

**Fig 1:**
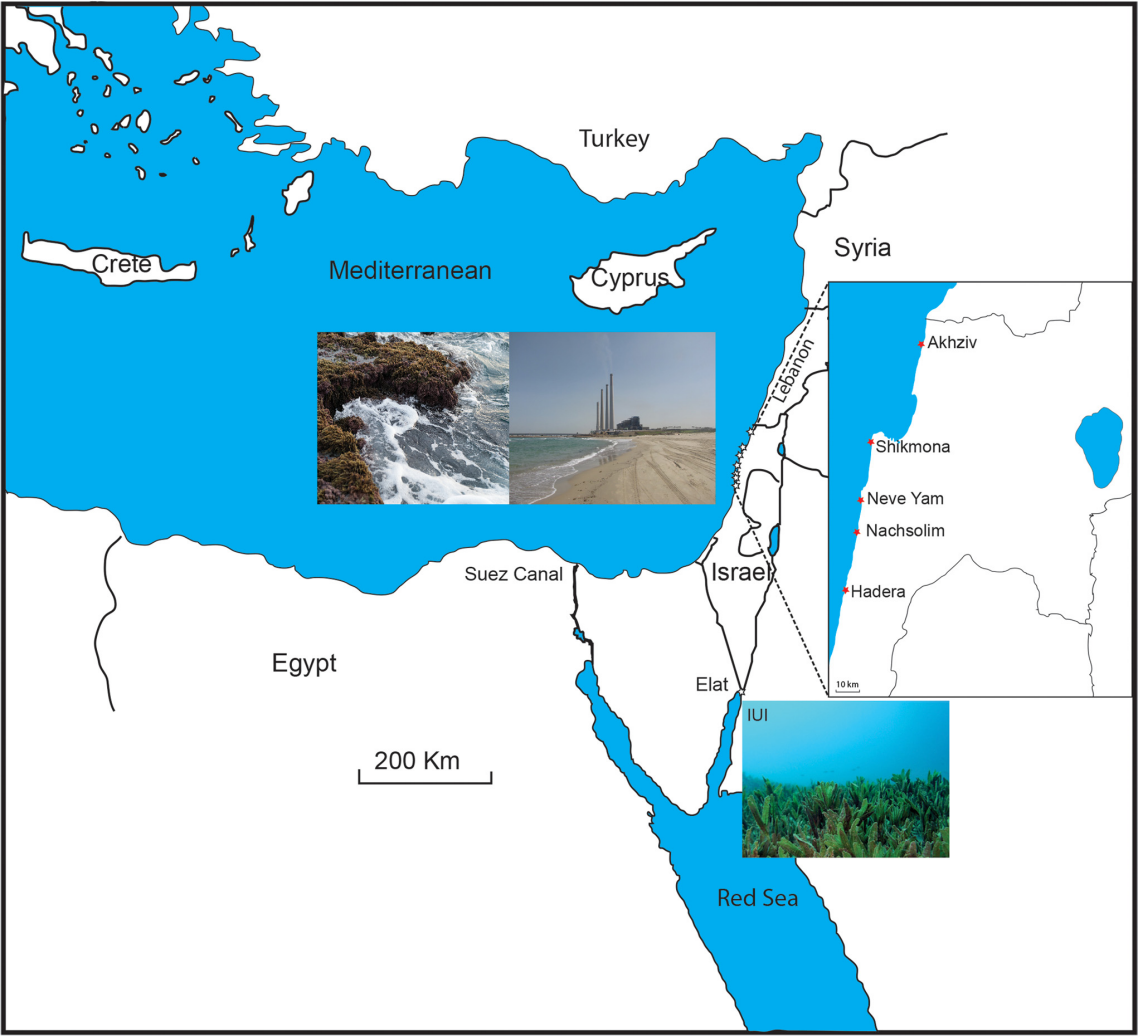
The location of the five sampling stations along the northern Israeli Mediterranean coast (Three stations in Hadera) and station IUI at the northern Red Sea. Upper right photo: View on station H2 near the “Orot Rabin” power plant in Hadera. Upper left photo: View on a hard bottom structure in Akhziv. Note the macroalgal cover and the strong wave action. Bottom photo: View on the *Halophila stipulacea* seagrass meadows at IUI. This figure is based on open street map data and is similar but not identical to the original image, and is therefore for representative purposes only.

The Eastern Mediterranean is a marginal, oligotrophic semi enclosed sea (Fig 1). The Israeli coast is part of the Levantine basin located at the eastern most part of the Mediterranean. This distal basin is known to be extremely oligotrophic, warm, and highly saline [36–38].

Natural hard bottom habitats (i.e. beach rocks, and abrasion platforms) are found throughout the northern Israeli coast, from intertidal to shallow neritic depths [39]. These habitats are typically characterized by a highly diverse marine ecosystem rich in macroalgae, soft body and calcareous organisms (e.g. gastropods, bivalves, calcareous algae). The latter make a significant contribution to the construction of biogenic crusts and other structures [40]. Organisms of these habitats are typically exposed to daily fluctuations of temperature and salinity, and are occasionally exposed to air at low tide (Fig 1).

**Fig 2:**
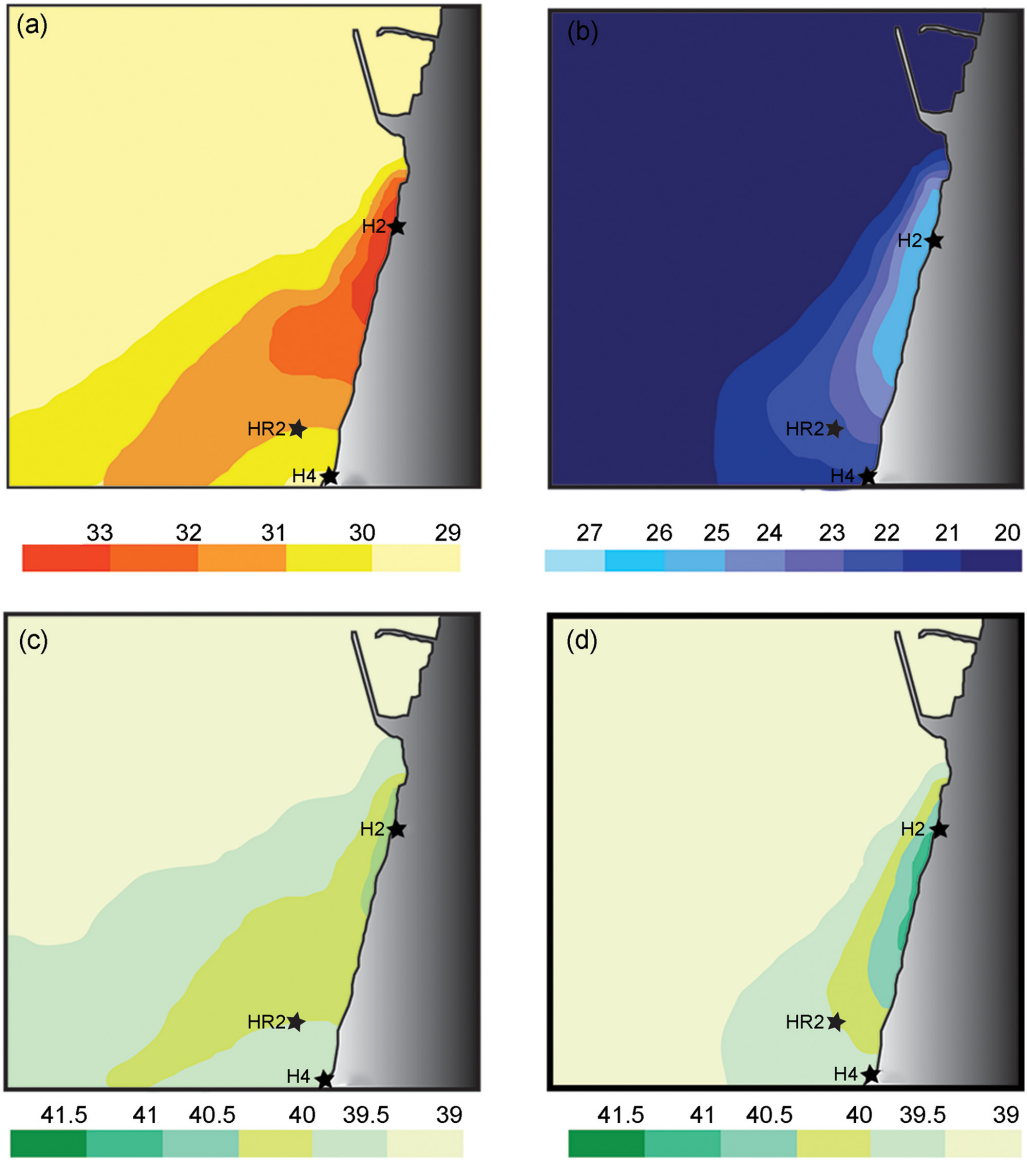
The spatial extent of temperature (a,b) and salinity (c,d) anomalies caused by heated water and brine discharge in Hadera during spring and fall months during 2010 (modified after [41]). This figure is based on open street map data and is similar but not identical to the original image, and is therefore for representative purposes only.

Living specimens of *T*. *agglutinans* were collected from seven intertidal hard bottom sites along the northern Israeli Mediterranean coast under the official approval of Israel Nature and Parks Authority (Fig 1, Table 1): Stations H2, H4, and HR2 are situated adjacent to the "Orot Rabin" power plant, and 4 natural, undisturbed intertidal hard bottom sites, Nachsholsim, Neve Yam, Shikmona, and Akhziv (Fig 2).

**Table 1:** Collection sites of *Textularia agglutinans* in the eastern Mediterranean Sea along the Israeli Mediterranean and Red Sea coasts.

Station Name	Water depth (meters)	Coordinates
Akhziv	0.2	33.056214° N, 35.102017° E
Shikmona	0.2	32.825368° N, 34.954788° E
Neve Yam	0.2	32.683152° N, 34.928052° E
Nachsholim	0.2	32.623422° N, 34.919611° E
Hadera H2	0.2	32.4585° N, 34.881167° E
Hadera HR2	5	32.4585° N, 34.881167° E
Hadera H4	0.2	32.447699° N, 34.879014° E
IUI, Elat	15 m	29 30.653 ° N, 034 55.454° E

The power plant "Orot Rabin" is active since the early 80's. It is located on the coast of the city of Hadera (Fig 2). Seawater is pumped year round into the power plant to cool the turbines and then discharged back into the sea via both the Hadera Stream and pipes. Since 2010 a desalination plant situated next to the power plant started to operate and added dilution of brines to the hot water stream. This creates a disturbed area that extends about 1.5 km to the south and 1 km to the west with unusually high temperatures of up to 36.5°C and salinity of up to 41‰. Station H2, is located right next to the hot stream outlet, representing the most disturbed sampling station. Station H4 is located about 1.5 km south from the outlet, where the plume influence is diminishing. Station HR2 is located about 1 km south from the outlet at a depth of 5 meters.

Water temperatures at Hadera (H2 and H4) and Nachsholim stations were recorded every 15 minutes from February 2013 to March 2014 using HOBO UA-002-08 temperature/light data loggers. This enabled us to document the temperature to which populations of *T*. *agglutinans* in these sites were exposed. The temperature data was daily averaged and statistically compared between stations using Kruskal-Wallis non parametric test and with Dunn's non parametric pairwise comparison.

#### The Gulf of Elat

The Gulf of Elat is a morpho-tectonic branch of the Red Sea, which is a part of the Syrian African rift system. Specimens examined for this study were collected from *Halophila stipulacea* seagrass meadows located near the Interuniversity Institute of Marine Sciences in Elat (IUI) at 15 meters water depth. In the Red Sea, *H*. *stipulacea* meadows are found from intertidal level, to about 70 m depth. Among the common inhabitants of *H*. *stipulacea* are various species of epiphytic benthic foraminifera, including *T*. *agglutinans*, which are found attached to all parts of the plant.

### Specimen collection for genetic analysis

**Fig 3:**
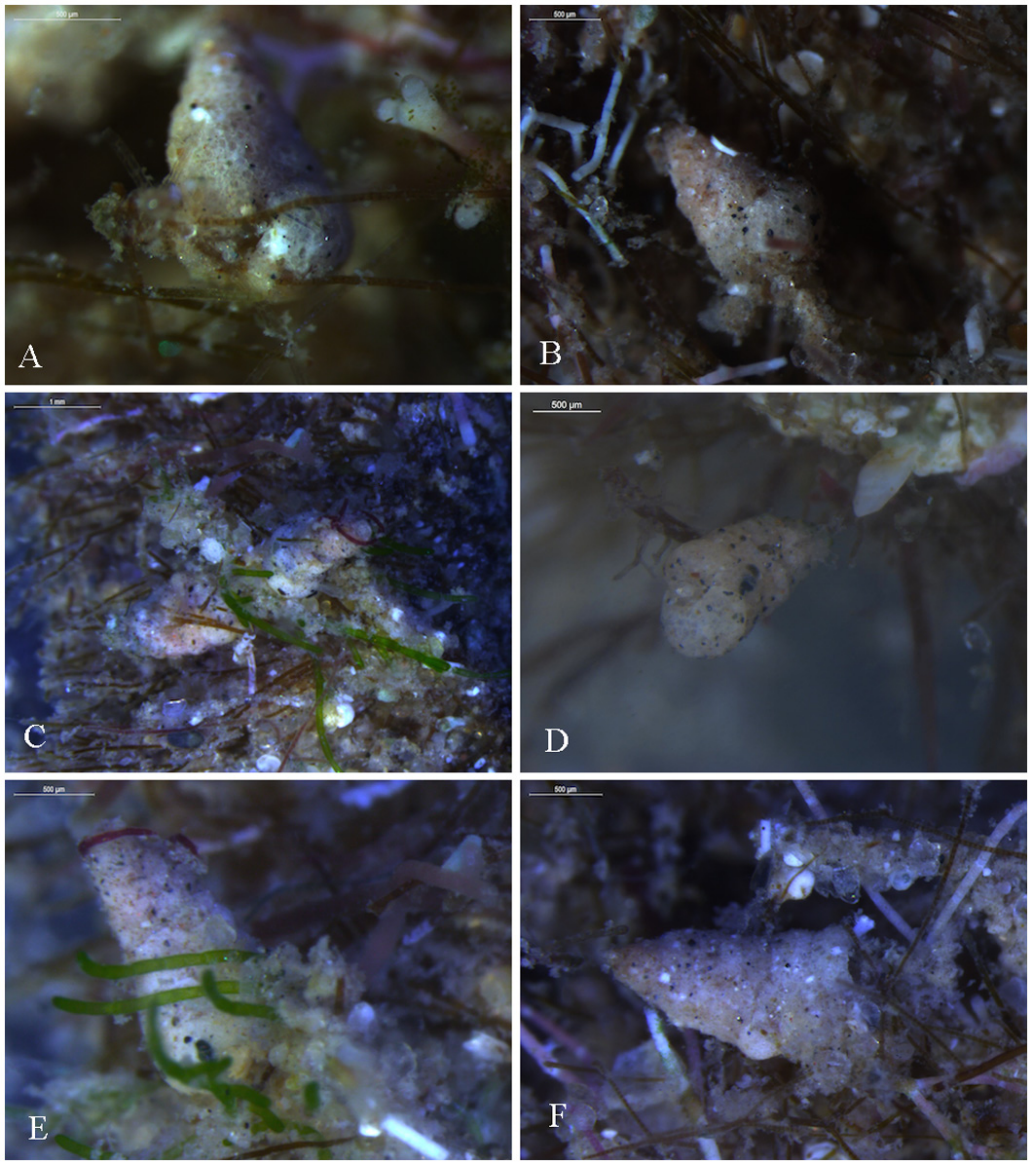
*Textularia agglutinans* epiphytic life mode from different stations. A-C, E-F. Shikmona; D- Neve Yam.

**Fig 4:**
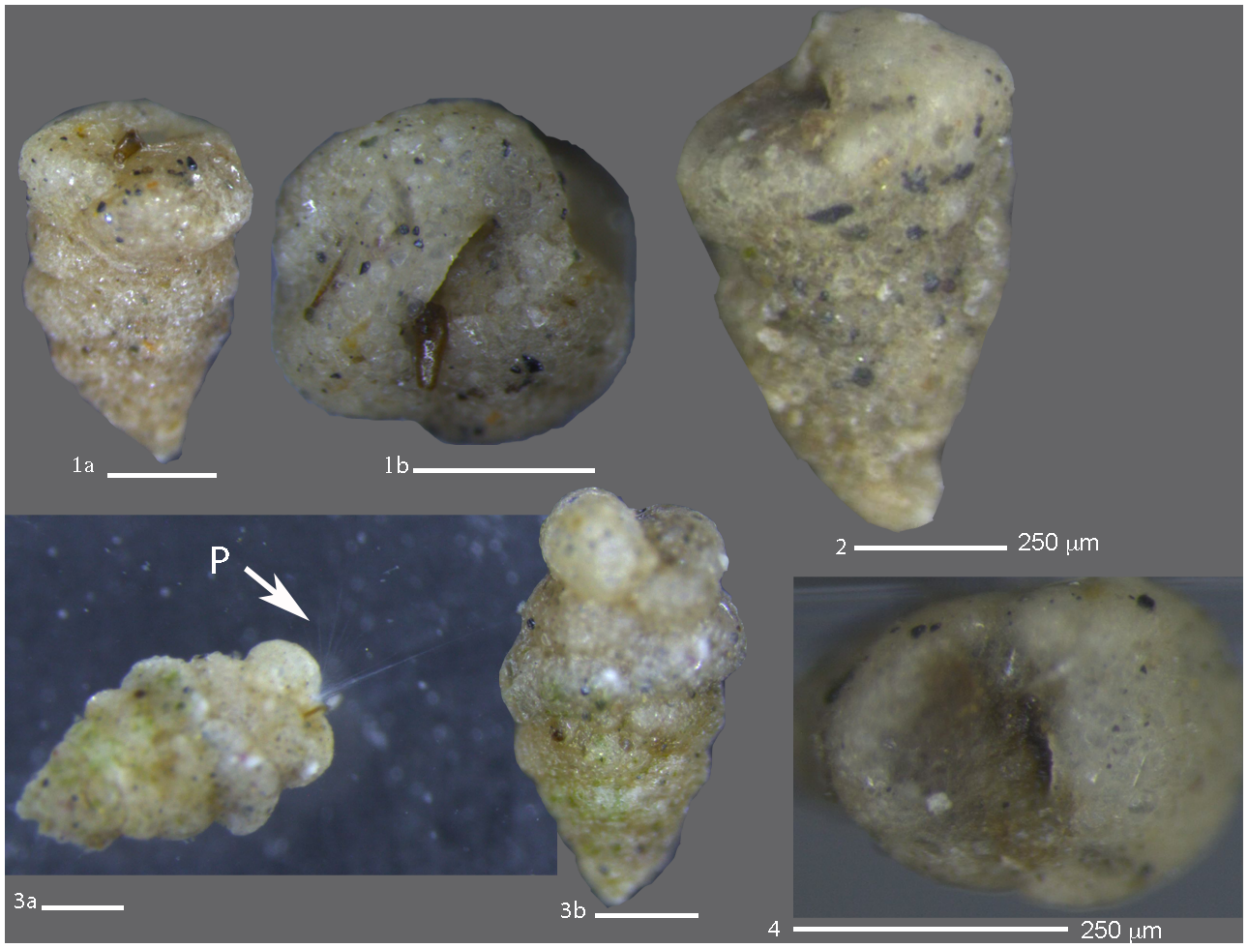
Living specimens of *Textularia agglutinans*. Living specimens of *T*. *agglutinans* from the Israeli Mediterranean shallow hard bottom environment, showing the variability in test and aperture shapes, and pseudopodia (P) of living specimens. Scale bars 100 μm except of specimens 2, 4 (250 μm).

**Fig 5:**
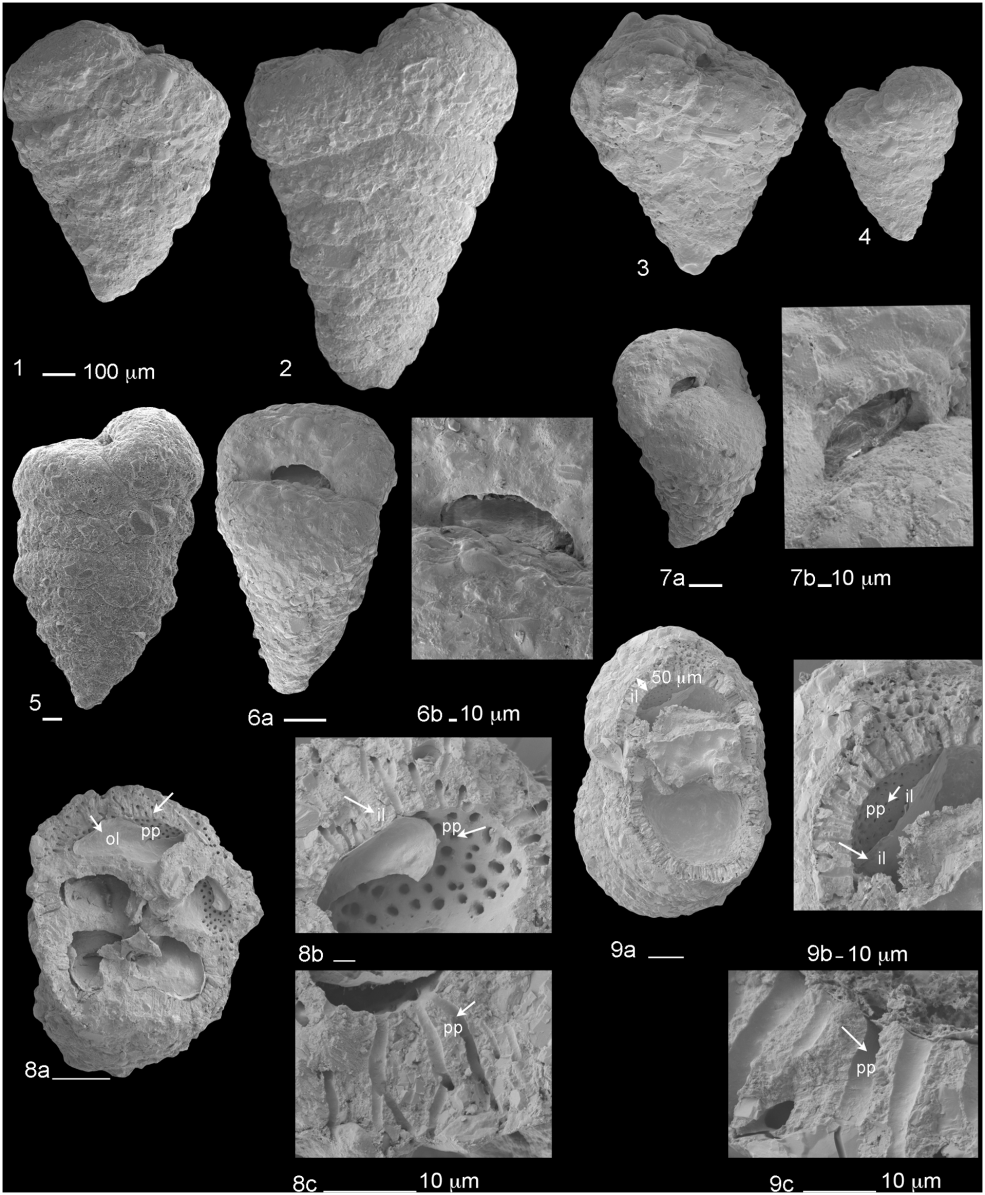
Scanning electron micrographs of *Textularia agglutinans*. 1–5. Lateral view (scale bars 100 μm); 6–7. Aperture view and a magnification of the aperture area, specimen 7 has a faint rim surrounding the aperture (7b) (scale of 6a and 7a 100 μm, scale bars of 6b and 7b 10 μm); 8–9. A perpendicular view to the long axis showing parapores (pp) and inner carbonate layer (il) with the organic layer (ol) blocking the parapores (scale bars of 8a, 8b, 9a 100 μm, scale bars of 8c, 9b, and 9c 10 μm).

Specimens of *T*. *agglutinans* were collected alive with the substrate they live on (turf and macroalgae, and seagrass) and transferred to containers with natural seawater (Fig 3). In the laboratory, living specimens were detected based on observations of pseudopodial extensions (Fig 4). The living specimens were cleaned of food remains by a delicate brush and photographed. The morphology of *T*. *agglutinans* has been documented using SEM (Scanning Electron Microscopy) and light microscopy with a digital camera (Leica, DFC290HD) (Figs 4 and 5). The width and length of 115 specimens was measured from digital photographs.

### DNA Extraction, amplification and sequencing

DNA extractions were performed on: 21 specimens of *T*. *agglutinans* from the shallow sites in the Israeli Mediterranean coast and 4 specimens from the Gulf of Elat, Red Sea. In addition, specimens of other textularids species from other locations were analyzed in order to evaluate the phylogentic position of *T*. *agglutinans* within this group. These include: *Textularia* sp. from Andaman Sea, Thailand and one specimen of *Textularia pseudogramen* from Faroe Island,.

DNA was extracted from single specimens using Qiagen DNeasy Plant Mini Kit. A fragment of the 3' end of the SSU rDNA (~900 bp) was amplified by semi nested PCR in two overlapping fragments. For the first PCR 1 μl of the purified DNA was used in a total volume of 25 μl. The primers S14F3-NewR were used for the first PCR and S14F1-newR were used for the second PCR (Table 2). The thermal cycles used for both PCR’s consisted of: 4 minutes at 95°C, 20 seconds at 98°C, 15 seconds at 65°C, and 1 minute at 72°C. The last cycle was followed by 5 minutes at 72°C for final elongation. For 10 specimens the Internal Transcribed Spacer region (ITS) was amplified in addition to the partial SSU. Primers S20-2TAIC were used for the PCR and thermal cycles were identical to those used for the partial SSU amplification.

**Table 2:** Primes used for DNA amplification.

Name	Sequence	Forward	Reverse
NewR	TTCATCGGTAAGAGCGAC		X
S14F1	AAGGGCACCACAAGAACGC	X	
S14F3	ACGCAMGTGTGAAACTTG	X	
S20	TTGTACACACCGCCCGTC	X	
2TAIC	CTCACTCGAGCTGATGTG		X

The amplified products were purified from gel and ligated into a pJET1.2 Plasmid using cloneJET PCR cloning kit. Ligation mix was transformed into DH5ɑ competent bacteria. Plasmids were extracted by Qiagen MiniPrep kit. For each specimen at least two clones were sequenced to screen for intra-individual variability.

The new sequences reported in this paper were deposited in the EMBL/GenBank database and their accession numbers are listed in Tables 3 and 4.

**Table 3:** Details on *Textularia* SSU sequences obtained in this study.

Isolate	Sampling locality	Species	Sampling depth (m)	No. of sequenced clones	Accession No.
322	Nachsholim	*T*. *agglutinans*	0.2	2	LN832472, LN832473
320	Nachsholim	*T*. *agglutinans*	0.2	1	LN832474
315	Nachsholim	*T*. *agglutinans*	0.2	2	LN832475, LN832476
316	Nachsholim	*T*. *agglutinans*	0.2	2	LN832477, LN832478
317	Nachsholim	*T*. *agglutinans*	0.2	2	LN832479, LN832480
318	Nachsholim	*T*. *agglutinans*	0.2	2	LN832481, LN832482
332	Nachsholim	*T*. *agglutinans*	0.2	1	LN832483
345	Hadera	*T*. *agglutinans*	0.2	2	LN832484, LN832485
346	Hadera	*T*. *agglutinans*	0.2	2	LN832486, LN832487
347	Hadera	*T*. *agglutinans*	0.2	2	LN832488, LN832489
348	Hadera	*T*. *agglutinans*	0.2	2	LN832490, LN832491
349	Hadera	*T*. *agglutinans*	0.2	2	LN832492, LN832493
361	Hadera (HR2)	*T*. *agglutinans*	5	1	LN832494
365	Hadera (HR2)	*T*. *agglutinans*	5	1	LN832495
367	Hadera (HR2)	*T*. *agglutinans*	5	2	LN832496, LN832497
368	Neve Yam	*T*. *agglutinans*	0.2	2	LN832498, LN832499
369	Neve Yam	*T*. *agglutinans*	0.2	2	LN832500, LN832501
370	Neve Yam	*T*. *agglutinans*	0.2	2	LN832502, LN832503
371	Neve Yam	*T*. *agglutinans*	0.2	2	LN832504, LN832505
447	Shikmona	*T*. *agglutinans*	0.2	1	LN832506
439	Akhziv	*T*. *agglutinans*	0.2	1	LN832507
13401	Thailand	*Textularia* sp.		1	LN848738
13402	Thailand	*Textularia* sp.		1	LN848739
13633	Faroe Islands	*Textularia pseudogramen*		2	LN848740, LN848741
17015	Gulf of Elat, Red Sea	*T*. *agglutinans*	15	3	LN879399, LN879400, LN879401
17016	Gulf of Elat, Red Sea	*T*. *agglutinans*	15	3	LN879402, LN879403, LN879404
17017	Gulf of Elat, Red Sea	*T*. *agglutinans*	15	1	LN879405
17019	Gulf of Elat, Red Sea	*T*. *agglutinans*	15	2	LN879406, LN879407

**Table 4:** Details on *Textularia agglutinans* ITS sequences obtained in this study.

Isolate	Sampling locality	Sampling depth (m)	No. of sequenced clones	Accession No.
317	Nachsholim	0.2	1	LN832553
318	Nachsholim	0.2	1	LN832545
332	Nachsholim	0.2	1	LN832547
361	Hadera (HR2)	5	1	LN832554
365	Hadera (HR2)	5	2	LN832549, LN832550
367	Hadera (HR2)	5	2	LN832551, LN832552
439	Akhziv	0.2	1	LN832546
447	Shikmona	0.2	1	LN832548

### Sequence analyses

**Fig 6:**
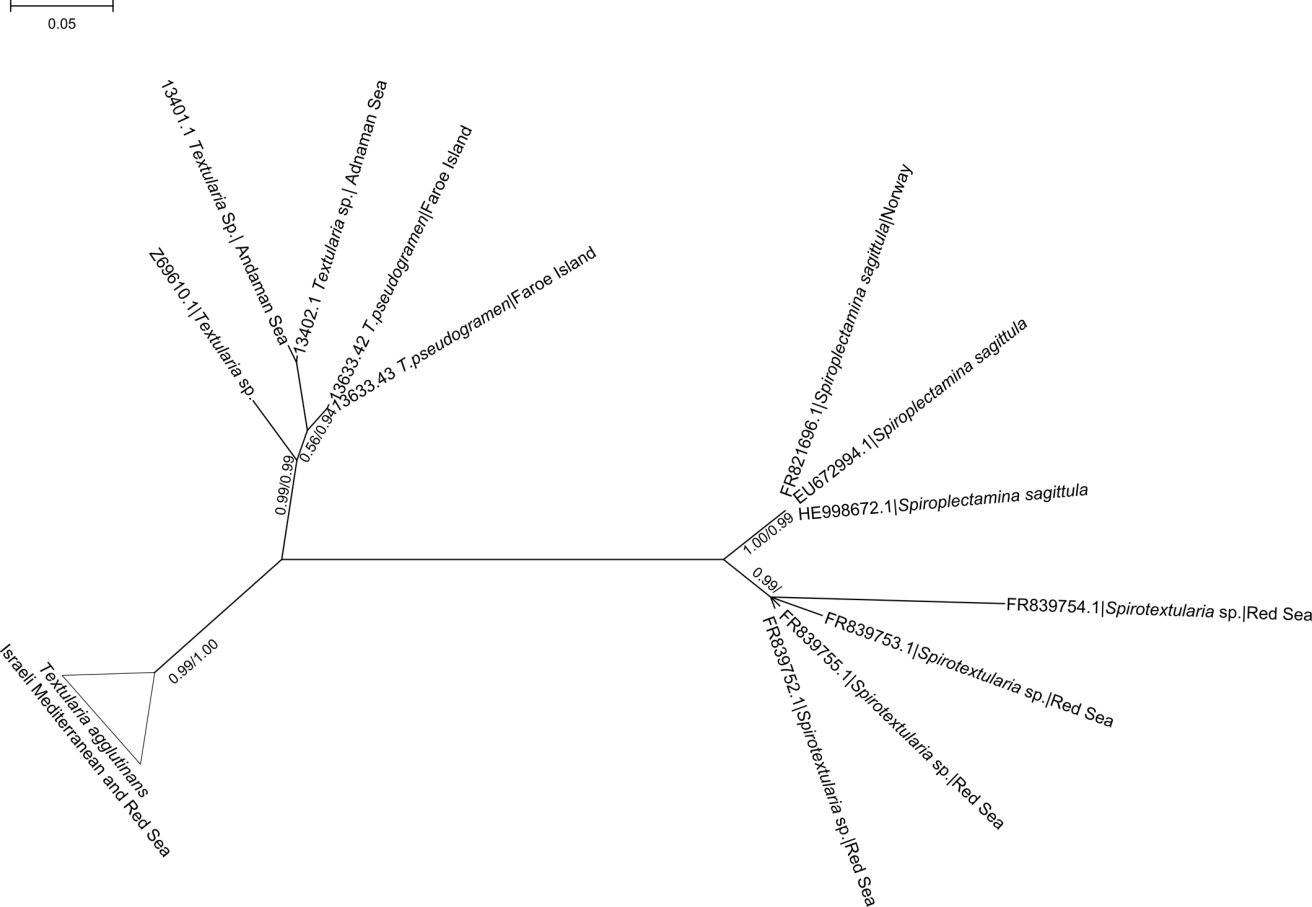
Unrooted phylogenetic tree of textularids species based on SSU rDNA. *Textularia agglutinans* from the Israeli Mediterranean and IUI, Red Sea are collapsed. Numbers at nodes indicate (from left to right) bootstrap values (ML) and posterior probability (BI). GenBank accession numbers are indicated on the tree as well as sampling locations when available.

**Fig 7:**
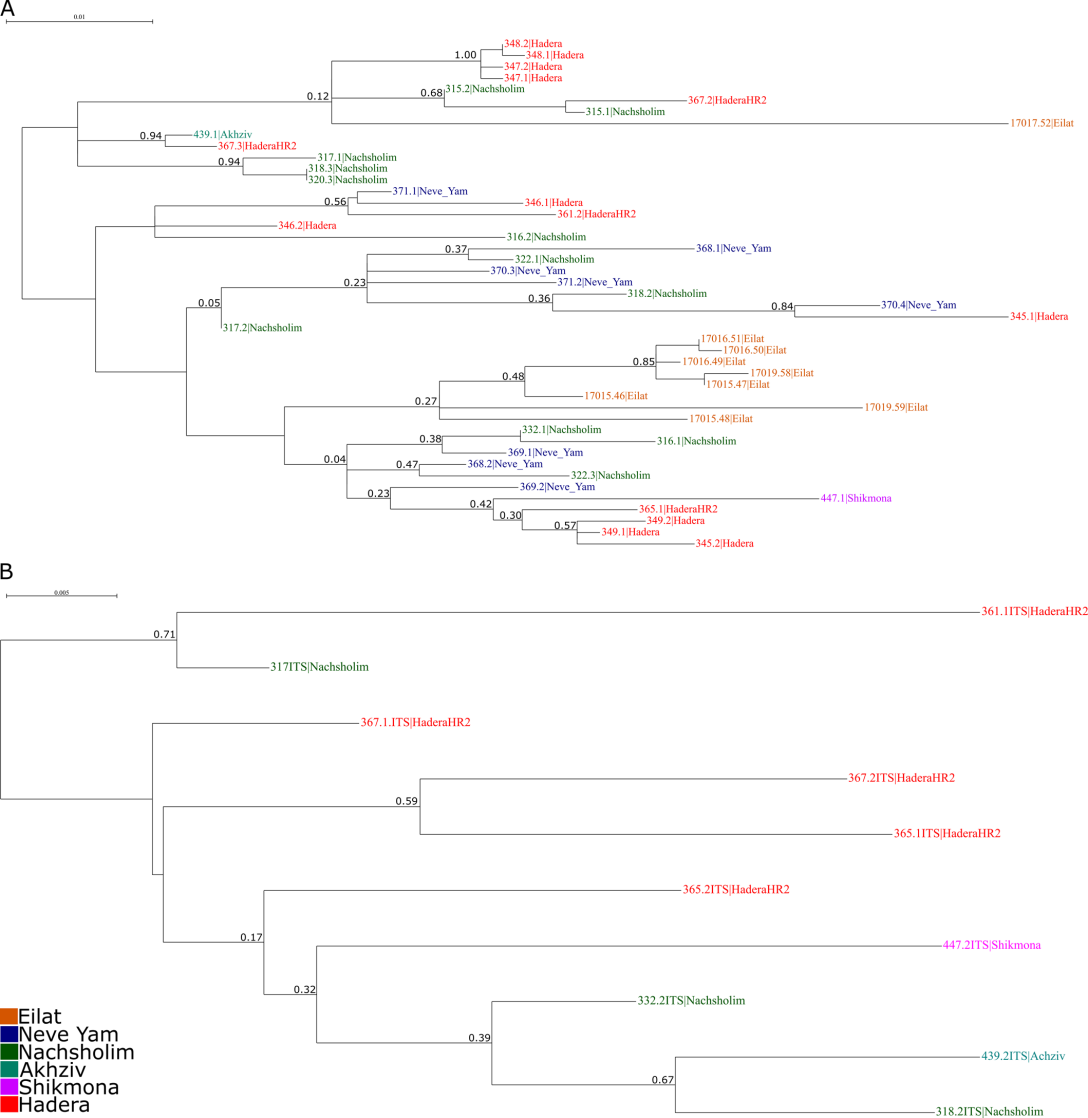
Unrooted phylogenetic trees of *Textularia agglutinans* from the Israeli Mediterranean and IUI, Red Sea. Colors indicate different sampling locations. A- Phylogenetic tree based on SSU rDNA. Numbers at nodes indicate (from left to right) bootstrap values (ML) and posterior probability (BI). B- Phylogenetic tree based on ITS sequences. Numbers at nodes indicate bootstrap values (ML).

The phylogenetic analysis was done using MEGA version 6 [42]. The sequences were automatically aligned by using the MUSCLE algorithm [43] with other textularids of the species *Spiroplectammina sagittula* and *Textularia pseudogramen*, and of the genus *Spirotextularia* from different locations published in NCBI GenBank (accession numbers are shown on the tree). Maximum likelihood analysis was based on the Tamura 3-parameter model [44]. Bayesian analysis was performed with MrBayes 3.2.4. The analysis consisted of 4 simultaneous chains that were run for 4,250,000 generations, and 12,752 trees were sampled, 3,188 of which were discarded as burn-in. Posterior probabilities at all nodes were estimated for the remaining trees (Fig 6). Another maximum likelihood analysis was performed with only the *T*. *agglutinans* SSU sequences from the Israeli Mediterranean in order to see the intra-specific variations of the SSU in *T*. *agglutinans* and with ITS sequences (Fig 7).

### Specimens counting

Quantitative data of *T*. *agglutinans* abundances were obtained from a conjoint ecological study on foraminiferal assemblages from the Mediterranean coast of Israel living in the Hadera heat patch (stations H2 and H4) and in Nachsholim, which was chosen as a control site, representing normal beach rock environment (Fig 1). Monthly sampling began in January 2013 and spanned 15 months (with the exception of station Nachsholim which started to be sampled from April, 2013). Triplicate samples of algal mats along with their entrapped sand were separated from the rocks with a sharp flat knife, placed in sampling containers and stained with Rose Bengal solution (2g of Rose Bengal per 1 liter of ethanol, following [45]) in order to mark the living foraminifera in the sample. From each sample, all stained foraminifera were picked to insure ideally at least 250 individuals from >63 μm size fraction. Specimens counting of *T*. *agglutinans* were determined based on their occurrences within these samples. The abundances were monthly averaged and statistically compared between stations using a Welch’s test with Tukey HSD post hoc test as an alternative to one-way ANOVA since the homogeneity of variances assumption was violated. The statistical analysis was performed using STATISTICA 10 software.

## Results

### Temperatures

**Fig 8:**
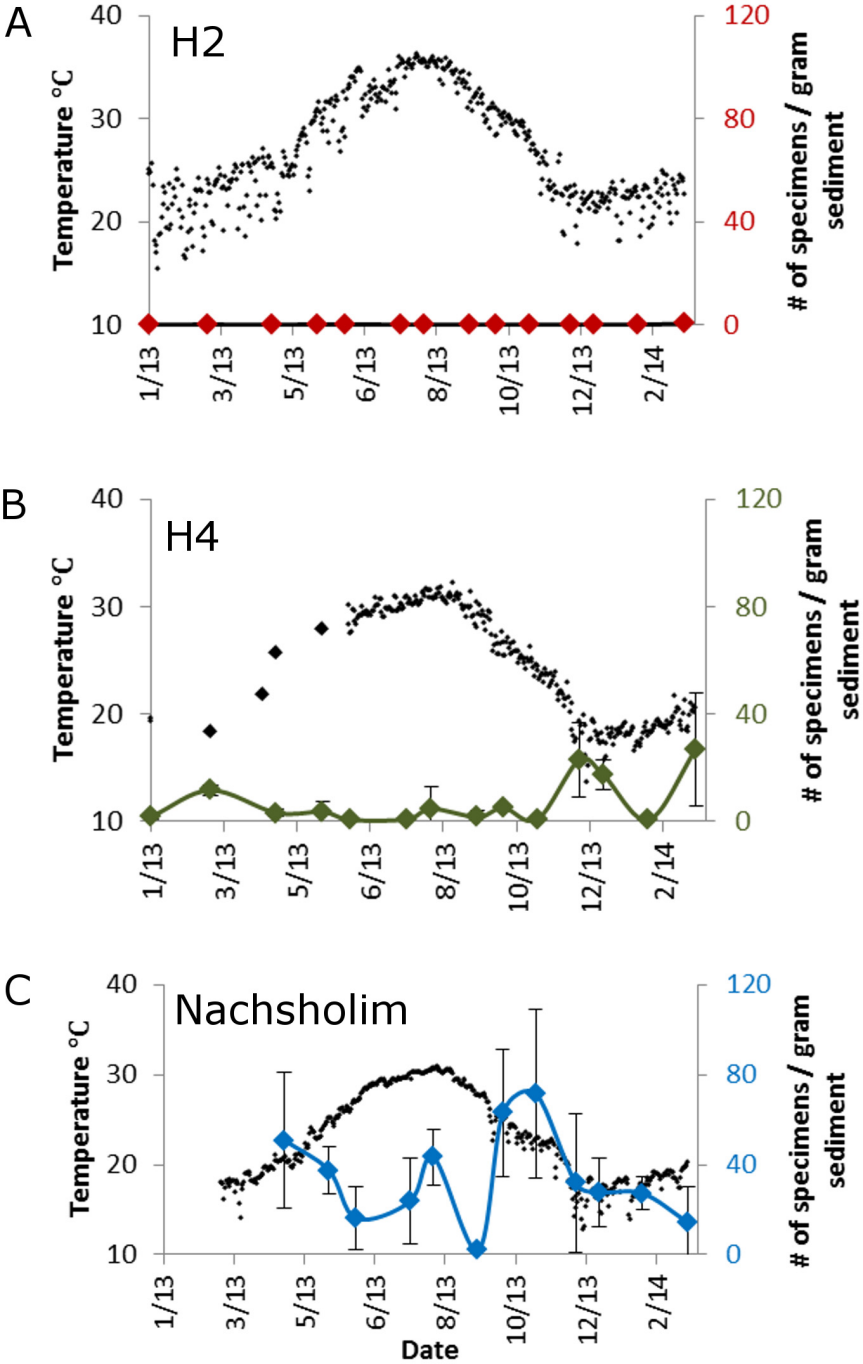
Abundance of *Textularia agglutinans* in # specimens / gram sediment in different stations. Station H2 (at the center of the Hadera heat plume), H4 at the edge of the heat plume with lower temperatures, and Nachsholim that experiences natural conditions and was used as a control station. Black dots indicate temperature measurements (left axis), colored line and diamonds indicate the average number of specimens per gram sediment from three replicates of each sample (right axis).

The heat patch anomaly and gradient are well reflected by the significant temperature differences between the warmest station H2 and the distant station H4, with lower temperature, all year round, and by the fact that temperatures of both stations were warmer than the natural control station Nachsholim (p<0.0001, Kruskal-Wallis/Dunn's test, Fig 8). Peak summer temperatures were recorded during August and September when maximum daily average temperatures reached 36°C in H2, 32°C in H4, and 31°C in Nachsholsim. Minimum daily average temperatures were recorded in January in station H2 (~15°C) and in December in stations H4 (14°C) and Nachsholim (13°C).

### *Textularia agglutinans* seasonal distribution

**Fig 9:**
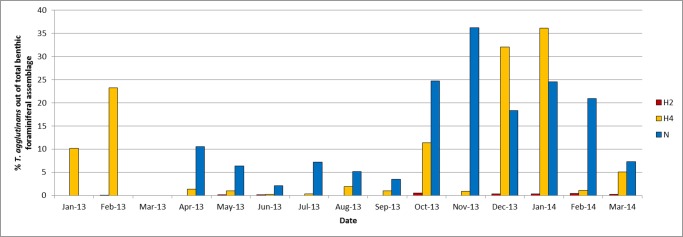
Percentage of *Textularia agglutinans* out of the benthic foraminiferal assemblage. *T*. *agglutinans* percentage in three stations: H2 at the center of the Hadera heat plume, H4 at the edge of the heat plume and Nachsholim—a control station with natural condition.

Apparently, the heat anomaly of the Hadera power plant has a profound effect on the occurrence of *T*. *agglutinans*, which exhibits a very low abundance within the plume, in the distant station H4, with some increase in winter. At the warmest station H2, *T*. *agglutinans* was rarely found throughout the year (0–0.4 specimens per gram sediment, Fig 8). In contrast, this species commonly occurs in the natural shallow beach rock habitat of Nachsholim, where its relative abundance varies between 2–70 specimens per gram sediment. Highest abundance was recorded at this station during fall and winter 2013, with two subordinate peaks occurring during spring and summer. The relative percentage of *T*. *agglutinans* was considerably higher during fall and winter (~35%) making it one of the most dominant species of the entire assemblage (Fig 9).

It is also apparent from our investigation of the natural hard bottom habitats along the Israeli Mediterranean coast that *T*. *agglutinans* has been well adjusted to epiphytic life mode in these habitats where it was commonly found attached to stalk or roots of coralline algae or turf’s algal substrate (Fig 3).

### Taxonomy

Phylum Foraminifera (d'Orbigny, 1826) [46]

Class Globothalamea Pawlowski, Holzmann and Tyszka 2013 [47]

Order “Textulariida” (Delage and Hérouard, 1896) [48]

Family Textulariidae Ehrenberg, 1838 [49]

Genus *Textularia* Defrance, 1824 [50]

*Textularia agglutinans* d'Orbigny, 1839 [1]

*Textularia agglutinans* d'Orbigny, 1839 [1] pl. 1 figs. 17, 18, 32–34, [Recent, Cuba, Saint Thomas]

*Textularia agglutinans* d'Orbigny, Brady, 1884 [51] pl. 43 figs. 1–2 [Recent, Challenger Station 135, Tristan da Cunha and off British territories from 183-274m depth]

*Textularia agglutinans* d'Orbigny, Le Calvez, 1977 [52], p. 13–14, fig. 1, [Recent, Cuba, Saint Thomas]

*Textularia agglutinans* d'Orbigny, Banner and Pereira 1981 [14], pl. 1, figs. 6–7, pl 2 fig. 1, [Recent, Coral reef, Mombasa]

*Textularia agglutinans* d'Orbigny, Cimerman and Langer 1991 [20], pl. 10, figs. 1–2. [Recent, Soft sediments, Mljet, Lake Valiko; Lončarić, Croatia]

*Textularia agglutinans* d'Orbigny, Hottinger et al. 1993 [7], pl. 13 figs. 1–9 [Recent, Gulf of Aqaba-Elat]

All specimens collected in this study from the shallow water sites along the Israeli Mediterranean and Red Sea coasts are morphologically classified as *T*. *agglutinans*. Explicitly, these forms morphologically resemble the specimens described by [7] and [14], and exhibit the main characteristic features of this species which includes a biserial agglutinated test with a carbonate inner layer penetrated by parapores canals (= canaliculi), a slit shape aperture, test narrowly triangular in lateral and peripheral view, periphery rounded, and sutures very slightly curved (Fig 5).

**Fig 10:**
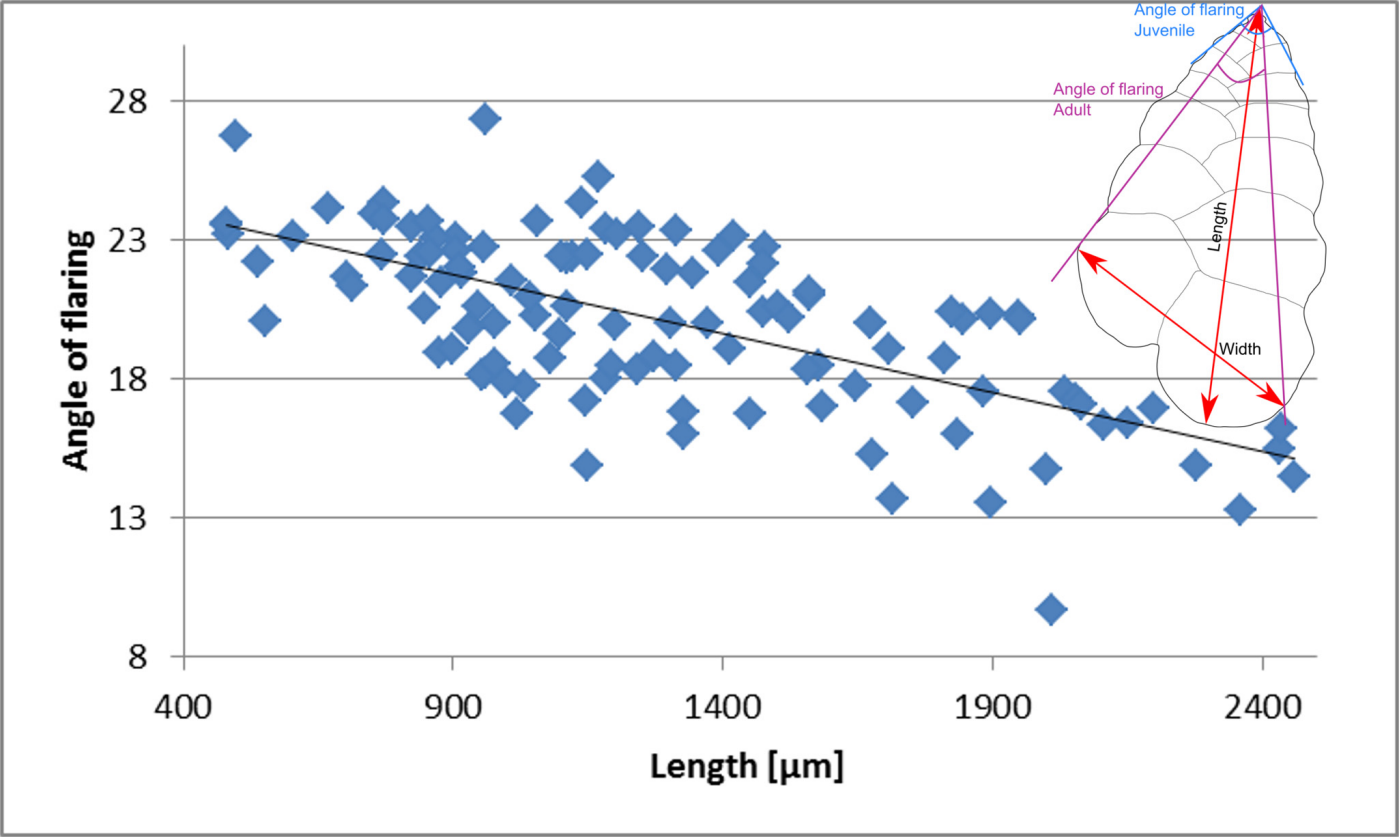
Measurements of angle of flaring as a function of test length. At early growth stage the chambers increase in size (width) rapidly while at later stages the chambers size stop increasing.

**Fig 11:**
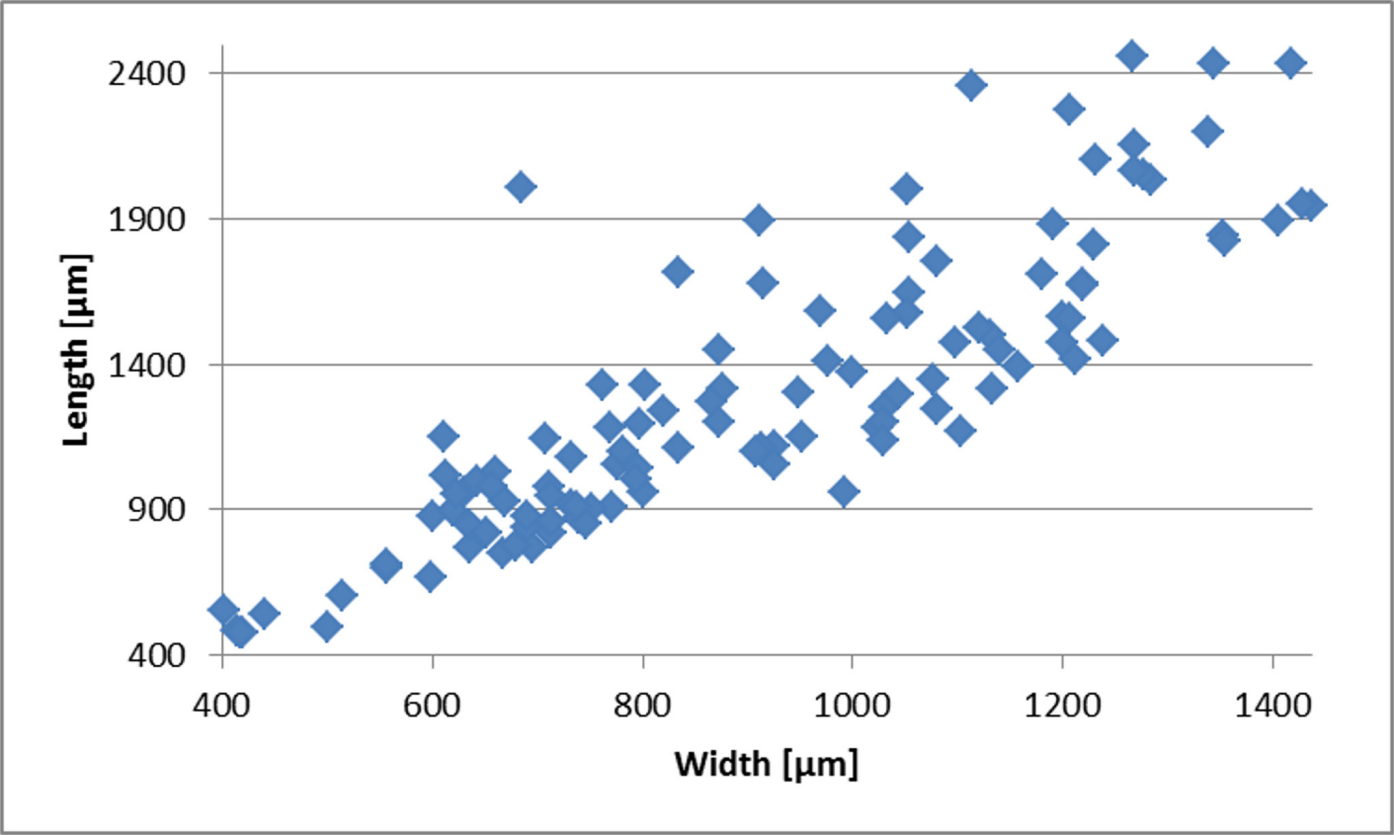
Measurements of length vs. width. Data from 115 *T*. *agglutinans* specimens collected in this study. The average Length:Width ratio is 1.4±0.3.

The most notable morphological variations among the examined material include differences in juvenile and adult flaring angles, test’s length, and degree of suture’s depression. The maximum length, width and the angle of flaring of the juvenile and adult stage were measured for 115 specimens of *T*. *agglutinans* from all sampling stations. The angle of flaring of the adult stage was measured between the first chamber and the periphery of the last two chambers in the widest position (see Fig 10 inset). The length and width of the collected specimens (juveniles and adults) vary between 480 μm– 2459 μm (average 1393.5 ±483 μm) and 402 μm– 1437 μm (average 913 ±264 μm) respectively. The length: width ratio varies between 0.97 and 2.93 with an average of 1.41 ±0.28 (Fig 11) which remains similar throughout ontogeny. [7] Reported a length:width ratio in the range of 2.1–2.6 for specimens from the Gulf of Aqaba-Elat (Red Sea), which is within the range we measured from the Mediterranean specimens. The variability of the length:width ratios observed in our specimens was found to be considerably larger than that measured by [7].

The angle of flaring varies between 13° up to 25° (Fig 10). Specimens with a wider flaring angle are relatively short (length less than 1500 μm). Whereas, the long forms (length > 1500 μm) exhibit a more acute flaring angle (~15°). Moreover, the latter also exhibit a distinct decrease in chambers growth rate in the adult stage. This change leads to a curved periphery and subparallel sides of the adult stage, observed in lateral view (Figs 4 and 5). In the shorter forms the chambers regularly increase in size as they are added. The degree of suture’s depression varies from slightly depressed to highly depressed sutures forming large rounded chambers, especially in the adult stage (Figs 4 and 5).

### Genetic analysis

The phylogenetic tree constructed from the obtained SSU sequences shows that all *T*. *agglutinans* specimens from the Israeli Mediterranean and from IUI, Red Sea cluster in a single clade (99% bootstrap value), well separated from other *Textularia* species and the other textularids genera, *Spirotextularia* and *Spiroplectammina* (Fig 6). The further divisions within this clade have very low bootstrap values (Fig 7a), suggesting that there is no apparent genetic division between the studied specimens of *T*. *agglutinans* from the different sites. Moreover, within *T*. *agglutinans*, the distance between some clones of the same specimen is greater than the distance between different specimens from two locations (Fig 7a), therefore representing an intra-genomic rather than inter-species variability. To support this observation, we analyzed the more rapidly evolving ITS rDNA region in 10 *T*. *agglutinans* specimens, from the Mediterranean sites. The phylogenetic tree, which was constructed from these sequences, does not show any clustering of specimens, thus further indicating that they all belong to a large population of a single genotype (Fig 7b).

## Discussion

### Ecological observations on *Textularia agglutinans*

*Textularia agglutinans* is a common agglutinated species which has been reported from all oceans. Previous reports indicate that it is present in almost every ocean from tropics to mid latitudes and at various water depths ranging from very shallow intertidal habitats to few hundred meters [15,18,26,27]. Yet the majority of these reports suggest that this species favors sandy to muddy surface sediments or even found as infaunal [22,23], although other species of the genus are known to be epiphytic and were reported from various types of algae [53,54]. It is important to note that the lack of reports of this species as an epiphyte might be due to lack of studies dealing with such habitats.

In recent years, this species became a very dominant component of the benthic foraminiferal assemblage along the Israeli Mediterranean rocky coast (e.g. [26]). Our ecological investigation shows that it is particularly dominant during the fall and winter as observed in the natural habitats of Nachsholim as well as within the Hadera heat plume at station H4 (Figs 8 and 9). Similarly, [26] reported peak abundances of *T*. *agglutinans* during winter at station H4 in Hadera, confirming our observations. High abundance of *T*. *agglutinans* was also reported in a recent survey of the shallow rocky habitats of the Israeli Mediterranean, where this species dominated the assemblages with *Amphistegina lobifera* and *Lachlanella* sp. [21]. Interestingly, this species was not found in fossil Quaternary shallow water records studied by [55], [56], and [57], but was recognized in Quaternary sediments from the Tyrrhenian Sea and in Turkey [58,59]. Moreover [60] in their survey on benthic foraminifera from the Israeli Mediterranean coast reported living *T*. cf *agglutinans* only from sandy mud at 30 meters water depth and not from the littoral habitats. The fact that *T*. *agglutinans* was not reported in the 1960’s coastal surveys and that its genetic sequences are identical to those of IUI red Sea (Fig 6) suggests that it is a new Lessepsian invader in the Israeli coast (see discussion below). [58,59]

Our study also reveals that in the intertidal hard bottom habitats of the Mediterranean coast of Israel, this species is fully epiphytic living on various types of macroalgae and turf complex, where it is typically found in remarkable high numbers (Figs 3 and 8). This particular life mode is most likely the key for the successful colonization of *T*. *agglutinans* on the hard bottom habitats along the Israeli Mediterranean coast.

The surface temperature in the eastern Levantine Basin varies naturally between 18°C in winter and 30°C in summer making this basin the warmest in the entire Mediterranean [61]. Over the past 44 years, an increase of 2°C has been recorded in the Eastern Mediterranean, most of which occurred since the 80’s [62]. This pattern implies that future warming in this region will cause a temperature rise well above 30°C in todays’ natural environments.

Our ecological survey indicates that such warming could have significant ramifications on the future spread of *T*. *agglutinans* in the eastern Levantine Basin. In the Hadera heat plume, where SST rise above 35°C in the summer, and exceeds the natural conditions by about 5°C, the abundance of this species is considerably lower than those recorded in the natural habitat of Nachsholim (Fig 8). This negative response is most prominent in the warmest station H2, where the abundance of *T*. *agglutinans* is less than 1 specimen per gram sediment indicating a low tolerance to SST > 35°C (Fig 8). The same response was reported by [26] that studied the Hadera plume in 2007 prior to the construction of the desalination plant. This clearly indicates that temperature is the major factor affecting the abundance of *T*. *agglutinans* in this substrate. Therefore, the successful colonization of *T*. *agglutinans* in the hard bottom habitats along the Israeli Mediterranean coast seems to be limited to natural habitats with normal temperature. Future rise in SST in this region is expected to have a negative effect on the occurrence of this species.

### Morphological variability within *Textularia agglutinans*

Generally, the morphological characteristics of *T*. *agglutinans* from previous reports and from our material are quite distinct (i.e., chambers arrangement, aperture shape, relatively large test size). However, the specimens collected in this study showed some morphological variability, especially in flaring angles, the test’s length, and the degree of suture’s depression (Figs 5, 10 and 11). This morphological variability does not correlate to the different localities or to environmental conditions (such as extreme heat). We have observed the entire morphological range in all stations throughout the year, which implies that these variations do not have a distinct ecophenotypic source, but rather reflect the natural variability within the population.

### Phylogeny of *Textularia agglutinans*

Since foraminifera-specific DNA extraction and amplification techniques were developed in the mid 1990’s [63], they provided new and unique opportunities to resolve phylogenetic relationships and examine the level of genetic similarity between species from different oceanic provinces (see more references in [64–68]).

Despite its common occurrences in all oceans, the genetic phylogeny and diversity of *T*. *agglutinans*, has not yet been studied. Moreover, until now, only 4 DNA barcoding sequences of textularids have been published (i.e. one of *Textularia* sp.; http://forambarcoding.unige.ch/, and 3 of *S*. *sagittula*; [47]), indicating a significant gap in our knowledge of the genetic taxonomy and phylogeny of this very common and important foraminiferal genus.

Our study presents the first genetic analysis of *T*. *agglutinans* that seems to have recently colonized the shallow hard bottom habitats along the Israeli Mediterranean coast. Our genetic results as shown in the partial SSU phylogenetic tree (Fig 6) demonstrate that all the specimens of *T*. *agglutinans* from the Israeli Mediterranean and Red Sea coasts, examined in this study, belong to a single haplotype.

These results indicate that the Red Sea population might be the source of the *T*. *agglutinans* found in the shallow hard bottom habitats along the Israeli Mediterranean and therefore this species should be considered as a Lessepsian invader (i.e. recent migration through the Suez Canal). Yet, we cannot exclude the possibility for the existence of other haplotype of *T*. *agglutinans*, which might represent different sources both in other localities and habitats in the Mediterranean and in the Red Sea.

No cryptic species were observed among the specimens from different localities. This observation is also supported by the ITS phylogenetic tree that shows that all examined specimens belong to a single haplotype and do not form clusters which correlate to morphology or to the different localities (Fig 7a). These results indicate that the morphological variations among specimens are taxonomically insignificant.

In many groups of foraminifera, cryptic species were discovered, which are genetically different from each other but have similar morphology (e.g. [66–68]). These records also confirm that the majority of benthic foraminiferal genotypes have a restricted geographic distribution. Indeed, the extraordinary worldwide distribution of *T*. *agglutinans* does raise the possibility of the existence of several genotypes in different regions and habitats. However, there are also several documented examples for extraordinary widespread species that share the same genotype. For example, several studies reported bipolar genetic similarity among several deep sea species including monothalamous forms [64,69]. Genetic similarity was also reported within genotype T1 of the genus *Ammonia* [70]. The question whether *T*. *agglutinans* is a widespread single species remains open, and requires a future comprehensive genetic and morphological analysis of specimens from the entire biogeographic range of this species.

Conclusions:

- The cosmopolitan species *Textularia agglutinans* recently colonized the hard bottom intertidal habitats along the Israeli Mediterranean coast, by having a fully epiphytic life mode.
- The successful establishment of this species in this area is indicated by its dominance in all natural hard bottom habitats throughout the Northern coastline of Israel, where it typically counted as the second most abundant species after *Lachlanella* sp. or *Amphistegina lobifera*.
- Genetic analysis of specimens of *T*. *agglutinans* from the Red Sea and Mediterranean coasts of Israel reveals that they belong to a single species, with no distinct differences between localities indicating the possibility of Lessepsian invasion.
- Ecological monitoring of a thermally impacted area of the Hadera power plant station documented extremely low abundance of *T*. *agglutinans* in the warmest stations, compared to a nearby undisturbed control station in Nachsholim. This pattern clearly indicates that this species cannot tolerate temperatures above 35°C. Our study suggests that the expected future rise in SST in the Levantine Basin will have harmful effect on *T*. *agglutinans*.
